# A PCNA-Derived Cell Permeable Peptide Selectively Inhibits Neuroblastoma Cell Growth

**DOI:** 10.1371/journal.pone.0094773

**Published:** 2014-04-11

**Authors:** Long Gu, Shanna Smith, Caroline Li, Robert J. Hickey, Jeremy M. Stark, Gregg B. Fields, Walter H. Lang, John A. Sandoval, Linda H. Malkas

**Affiliations:** 1 Department of Molecular & Cellular Biology, Beckman Research Institute of City of Hope, Duarte, California, United States of America; 2 Department of Molecular Pharmacology, Beckman Research Institute of City of Hope, Duarte, California, United States of America; 3 Department of Radiation Biology, Beckman Research Institute of City of Hope, Duarte, California, United States of America; 4 Torrey Pines Institute for Molecular Studies, Port St. Lucie, Florida, United States of America; 5 Department of Surgery, St. Jude Children's Research Hospital, Memphis, Tennessee, United States of America; University of Minnesota, United States of America

## Abstract

Proliferating cell nuclear antigen (PCNA), through its interaction with various proteins involved in DNA synthesis, cell cycle regulation, and DNA repair, plays a central role in maintaining genome stability. We previously reported a novel cancer associated PCNA isoform (dubbed caPCNA), which was significantly expressed in a broad range of cancer cells and tumor tissues, but not in non-malignant cells. We found that the caPCNA-specific antigenic site lies between L126 and Y133, a region within the interconnector domain of PCNA that is known to be a major binding site for many of PCNA's interacting proteins. We hypothesized that therapeutic agents targeting protein-protein interactions mediated through this region may confer differential toxicity to normal and malignant cells. To test this hypothesis, we designed a cell permeable peptide containing the PCNA L126-Y133 sequence. Here, we report that this peptide selectively kills human neuroblastoma cells, especially those with *MYCN* gene amplification, with much less toxicity to non-malignant human cells. Mechanistically, the peptide is able to block PCNA interactions in cancer cells. It interferes with DNA synthesis and homologous recombination-mediated double-stranded DNA break repair, resulting in S-phase arrest, accumulation of DNA damage, and enhanced sensitivity to cisplatin. These results demonstrate conceptually the utility of this peptide for treating neuroblastomas, particularly, the unfavorable *MYCN*-amplified tumors.

## Introduction

Neuroblastoma (NB) is one of the most common childhood neoplasms which originates from neural crest progenitor cells of the sympathetic nervous system and accounts for about 15% of all pediatric cancer deaths [1]. The prognosis of NB patients depends on the risk stratification. Survival is excellent in low and intermediate risk groups and localized perinatal adrenal tumors often spontaneously regress [2]. In contrast, patients in the high-risk group have very aggressive disease and represents a significant clinical hurdle [3]. Modern treatment for high-risk NB consists of induction treatment (conventional chemotherapy and surgery with or without radiotherapy), high-dose chemotherapy and autologous stem cell transplantation (HDCT/autoSCT) as a consolidation treatment, and 13-*cis*-retinoid acid to reduce relapse from minimal residual disease. Despite aggressive therapy, approximately 50% of patients with advanced disease are refractory to treatment or relapse and the survival rate for high-risk NB patients is dismal [4], [5]. Thus, there is a significant unmet medical need for new therapies to improve the treatment outcomes of this aggressive tumor phenotype.

Proliferating cell nuclear antigen (PCNA) is an evolutionally conserved protein found in all eukaryotic cells. It is an extensively used tumor progression marker, due to its function in DNA replication and its peak expression during S and G2 phases [6]–[8]. PCNA forms a homotrimeric ring structure encircling DNA [9], [10] and acts as a sliding clamp that provides anchorage for many proteins [11]. A major interaction site in PCNA is the interdomain connecting loop that spans from amino acid M121 to Y133 [9]. This loop is recognized by proteins including p21 (CDKN1A) [12], DNA polymerase ä (Pol ä) [13], flap endonuclease 1 (FEN1) [14], DNA methyltransferase MeCTr [15], and DNA ligase 1 (LIGI) [16], which interact with PCNA through their PIP-box domains [11], [17]. By recruiting these proteins to chromatin, PCNA plays a critical role in regulating DNA replication, cell cycle progression, and DNA damage responses [18]. Given the essential role of PCNA in coordinating these cellular processes that are fundamental to the proliferation and survival of cancer cells, inhibition of PCNA is viewed as an effective way to suppress tumor growth [19]. Extensive structural studies have enabled blockade of PCNA interaction by rational designs [20]–[22]. Several attempts have been made in recent years to block various aspects of PCNA function with promising results [12], [23]–[27], demonstrating the potential of PCNA as a target for anti-cancer therapies.

We previously reported a novel cancer associated PCNA isoform (caPCNA) [28], which is present in a broad range of cancer cells and tumor tissues, but not highly expressed in non-malignant cells. We determined that caPCNA arises not because of a genetic mutation but as a result of posttranslational modification [29]. caPCNA actively participates in DNA replication and interacts with cellular DNA polymerases [28]. Epitope analysis using monoclonal antibodies raised against caPCNA reveals that the caPCNA-specific antigenic site lies between L126 and Y133 within the interconnector domain of PCNA ([28] and to be reported elsewhere). Using an *in vitro* Biacore assay, we observed that the peptide corresponding to L126-Y133 (caPep) can block the PCNA interaction with the PIP-box sequence of FEN1. Interestingly, the L126-Y133 region is only accessible to immunohistochemistry staining by a monoclonal antibody specific to this region in tumor cells, suggesting that this region is structurally altered and becomes more accessible for protein-protein interaction in tumor cells. We hypothesized that therapeutic agents targeting protein-protein interaction mediated through this peptide region may confer differential toxicity to normal and malignant cells. To test this hypothesis, we designed a cell permeable peptide containing the L126-Y133 sequence of PCNA (R9-caPep, see Materials and Methods). Here, we report that this peptide selectively kills NB cells with much less toxicity to human peripheral blood mononuclear cells (PBMC) or neural crest stem cells. R9-caPep also suppressed NB cell growth in a mouse xenograft model. Interestingly, *MYCN*-amplified NB cell lines are more sensitive to R9-caPep treatment than non-*MYCN*-amplified lines. Mechanistically, R9-caPep is able to selectively block PCNA interactions in cancer cells. It interferes with DNA synthesis and homologous recombination (HR) mediated double-stranded DNA break (DSB) repair, resulting in S-phase arrest, accumulation of DNA damage, and enhanced sensitivity to cisplatin. These results demonstrate that targeting protein-protein interactions involving the L126-Y133 region of PCNA may prove to be an effective approach to treating high-risk *MYCN*-amplified NB patients with reduced side effects.

## Materials and Methods

### Peptides

The eight amino acid caPeptide (caPep) corresponds to the L126-Y133 sequence of human PCNA. The cell permeable peptide, R9-caPep (R_D_R_D_R_D_R_D_R_D_R_D_R_D_R_D_R_D_CCLGIPEQEY) was created by fusing the caPep to the C-terminus of a nine D-arginine sequence (R9) through a spacer of two cysteines (CC). Peptides containing nine D-arginines and two cysteines only (R9-CC) or the R9-CC sequence fused to the same amino acid residues as in the PCNA L126-Y133 region, but in a scrambled order (R9-srbPep: R_D_R_D_R_D_R_D_R_D_R_D_R_D_R_D_R_D_CCEPGLIYEQ) were synthesized as controls. 5/6-Fluorescein (5-FAM) labeled R9-caPep and R9-srbPep were utilized for fluorescence microscopy and FACS analysis. All peptides were synthesized by AnaSpec (Fremont, CA).

### Kinetic analysis of PCNA interaction by surface plasmon resonance (SPR)

All experiments were conducted on the Biacore T100 (GE Healthcare Life Sciences, Piscataway, NJ) in the running buffer HBS-EP+ (10 mM HEPES pH 7.4, 0.15 M NaCl, 3 mM EDTA, 0.05% v/v Surfactant P20). FEN1 peptide containing the PIP-box (SKSRQGSTQGRLDDFF) was immobilized on a carboxymethylated dextran modified CM5 chip using carbodiimide covalent linkage procedures outlined by the manufacturer (GE Healthcare Life Sciences). Recombinant PCNA (rPCNA), purchased from Surmodics, Inc. (Edina, MN), were serially diluted in the HBS-EP+ buffer and flowed over the FEN1-coated sensor chip in the presence of 0, 500, or 1000 nM of caPep at a 5 ìl/min flow rate with a contact time of 3 minutes, followed by dissociation under the same buffer condition and regeneration of the chip surface in 10 mM glycine-HCl (pH 2.0). Binding curves were recorded for rPCNA concentrations ranging from 250 to 1000 nM in the presence of 0, 500, or 1000 nM of caPep. Kinetic parameters from Biacore binding data were determined using Biacore T100 Evaluation Software Version 2.0.3.

### Cell permeability analysis

Cells were treated by various concentrations of 5-FAM labeled R9-caPep or R9-srbPep for 6 h. After being washed twice by PBS and detached by trypsin treatment, cells were further washed twice by the cell culture medium. The uptake of the fluorescent peptides was measured by a FACS analysis. The median fluorescent intensity was determined by the FlowJo program for each cell population under different treatment conditions. In addition, cells treated by 10 µM 5-FAM labeled R9-caPep or R9-srbPep were examined by confocal microscopy to determined the subcellular localization of the peptides.

### Plasmids and Cell Lines

The human NB cell lines, SK-N-DZ, SK-N-BE(2)c, SK-N-AS, SK-N-SH, and SK-N-FI were obtained from the American Type Culture Collection (Rockville, MD). Cells were maintained in DMEM with 10% fetal bovine serum (FBS), 100 units/ml penicillin, and 100 µg/ml streptomycin in the presence of 5% CO_2_ at 37°C. Human PBMCs from a healthy donor were purchased from Sanguine BioSciences (Valencia, CA) and grown in RPMI1640 with10% FBS, 100 units/ml penicillin, 100 µg/ml streptomycin, and 10 ng/ml IL-2 in the presence of 5% CO_2_ at 37°C. Human embryonic progenitor cell line 7SM0032 was acquired from Millipore (Billerica, MA) and grown in the hEPM-1 Media Kit purchased from the same company.

The plasmid pCBASce expresses the rare cutting I-SceI meganuclease [30]. The U2OS-derived cell lines, DR-GFP, EJ5-GFP, and SA-GFP contain a stably transfected reporter gene for DSB repair mediated by HR, end joining (EJ), and single-strand annealing (SSA), respectively [31]. These cell lines were cultured in DMEM medium with 10% FBS at 37°C in the presence of 5% CO_2_.

### Cell growth and terminal deoxynucleotidyl transferase–mediated dUTP nick end labeling (TUNEL) assays

To measure cell growth, cells were seeded at 3×10^4^/ml. After being allowed to attach overnight, cells were treated with various concentrations of the peptides for 72 h. Cell growth was measured by the CellTitor-Glo assay (Promega, Madison, WI) according to manufacturer's instruction. To measure apoptosis, cells were seeded at 1×10^5^/ml onto a chamber slide. Once attached, cells were treated with the peptides for 48 h. Cells were fixed and analyzed by a TUNEL assay using the TMR red *in situ* cell death detection kit (Roche Diagnostics, Indianapolis, IN).

### Cell Cycle Analysis

Cells were seeded at 1×10^5^/ml. Once attached, cells were treated with or without R9-caPep for 48 hours. Cells were fixed in 60% ethanol and stained with propidium iodide (PI). The cellular PI fluorescence intensity was determined by flow cytometry. The flow cytometry data were analyzed by the FlowJo program to model various cell populations.

### Immunofluorescence

Cells were seeded at 1×10^5^/ml onto a chamber slide and were allowed to attach overnight. To analyze the interaction of PCNA with FEN1, LIGI, or Pol ä, we first synchronize cells at the G1/S boundary. The synchronization is achieved by starving cells in medium containing 0.25% FBS for 24 h. Cells were further cultured in the complete medium containing 400 µM of mimosine for 24 h. To release cells into S phase, cells were washed and incubated in mimosine-free medium containing 30 µM R9-caPep or R9-srbPep for 6 h. We pre-determined that the majority of cells were in the S-phase 6 h after mimosine was removed (data not shown). Cells were fixed in ice-cold methanol:acetone (50%:50%) for 10 min or in 4% paraformaldehyde for 20 min at room temperature. Cells were incubated with a goat polyclonal anti-PCNA antibody (Santa Cruz) and a mouse monoclonal anti-FEN1 antibody (Santa Cruz), a mouse anti-POLD3 antibody (Sigma, St. Louis, MO), or a mouse anti-LIGI antibody (Abcam, Cambridge, MA) for 1 h at room temperature. After being washed with PBS, cells were incubated with Alexa Fluor 488 conjugated anti-mouse IgG and Alexa Fluor 555 conjugated anti-goat IgG antibodies (Invitrogen, Grand Island, NY) for 1 h. Cells were mounted in Vectashield with DAPI (Vector Labs, Burlingame, CA) and visualized by a confocal microscope.

To study DNA damage and repair, attached cells were pretreated with the peptides for 2 h and were then ã-irradiated (5 Gy). After irradiation, cells were cultured in the presence of the peptides for the indicated time. For analyzing ãH2A.X foci formation, cells were fixed in a solution of methanol and acetone (70%:30% v/v) for 15 min at −20°C. The slides were air-dried for storage and rehydrated in PBS prior to immunostaining. Cells were stained by a mouse monoclonal antibody specific to ãH2A.X (Millipore, Billerica, MA) followed by an Alexa Fluor 488 conjugated anti-mouse IgG antibody. For analyzing Rad51 foci formation, cells were fixed in PBS buffered 4% paraformaldehyde at room temperature for 15 min. After being washed twice by PBS, cells were permeabilized in PBS containing 0.5% triton for 15 min on ice. The fixed and permeabilized cells were stained with a rabbit polyclonal antibody raised against the human Rad51 (Santa Cruz) followed by an Alexa Fluor 488 conjugated anti-rabbit IgG antibody. Stained cells were visualized and imaged by a confocal microscope.

### BrdU incorporation assay

SK-N-BE(2)c cells were treated with the peptides for 7.5 h and then incubated in 10 ìmol/L BrdU for an additional 30 min in the continuous presence of the peptides. Cells were detached with trypsin and fixed in Cytofix/Cytoperm buffer according to the manufacturer's instructions (BD Bioscience, San Jose, CA). Fixed cells were treated with DNase to expose incorporated BrdU and stained with FITC-conjugated anti-BrdU antibody (BD Bioscience) for 1 h at room temperature. Samples were analyzed by flow cytometry to quantify the amount of BrdU incorporation. Percentages of FITC-positive cells were determined by analysis with the FlowJo software. Statistical analysis was conducted using a two-tailed t-test.

### SV40 DNA replication assay

The assay was performed essentially as described [32], except that nuclear extracts from SK-N-BE(2)c cells were used in the assay.

### DSB repair assays

DR-GFP, EJ5-GFP, and SA-GFP cell lines were seeded at 2.5×10^4^ cells/cm^2^ in a 12-well plate. Once attached overnight, cells were transfected with 1.2 µg of the pCBASce I-SceI expression plasmid mixed with 3.6 µl of Lipofectamine 2000 (Invitrogen) in 200 µl of Optimem media (Invitrogen). After incubation for 3 h, the media containing transfection complexes was aspirated and replaced with fresh media containing 30 µM of the peptides. The HR, EJ, and SSA-mediated DSB repair, indicated by the restoration of a functional GFP gene in the respective cell lines, were quantified by measuring the relative abundance of GFP-positive cells by flow cytometry 3 d after transfection.

### Clonogenic Assay

Three hundred fifty SK-N-BE(2)c cells were seeded onto a 60-mm tissue culture dish. Once attached overnight, cells were treated with or without cisplatin for 2 h. Cells were washed twice with growth medium and were cultured in fresh medium with or without R9-caPep for 3 weeks to allow colony formation. The medium was changed every 3 d. The colonies formed under each treatment conditions were counted after being stained with 0.5% crystal violet.

### Western blot

Cell extracts were prepared by dissolving cell pellets directly into the Laemmli sample buffer and resolved in a 4–12% SDS polyacrylamide gel. Resolved proteins were blotted onto a nitrocellulose membrane. The membrane was blocked with 5% nonfat dry milk and incubated with an antibody specific to ãH2A.X (Millipore), total H2A.X (Cell Signaling Technology, Danvers, MA), MYCN (Cell Signaling Technology), or actin (Sigma) in the blocking buffer. After incubation with peroxidase-conjugated secondary antibodies, the protein of interest was detected using an ECL kit purchased from GE Healthcare.

### *In vivo* tumor model

All experiments involving live animals were carried out in strict accordance with the recommendations in the Guide for the Care and Use of Laboratory Animals of the National Institutes of Health. The protocol (#11034) was reviewed and approved by the City of Hope Institutional Animal Care and Use Committee. Nude mice 6 weeks of age were purchased from the Jackson Laboratory (Bar Harbor, ME). SK-N-BE(2)c cells were harvested and washed twice in PBS. Cells were suspended in Matrigel (BD Biosciences) at 5×10^7^/ml. 0.1 ml of suspended cells was subcutaneously injected into the right flank of each of 30 nude mice. Seven days after tumor inoculation, mice were randomly grouped into three groups with 10 mice in each group. The mice were treated with PBS, R9-srbPep, or R9-caPep 3 times a week by intratumoral injection. Tumor growth was measured weekly as well as at the end of the experiment by a dial caliper. Tumor volumes were estimated based on the length (L) and width (W) of the tumors (V = L×W^2^×0.5). At the end of the experiment, tumors were isolated from sacrificed mice and their masses were measured.

## Results

### Cell permeable R9-caPep selectively inhibits the growth of NB cells

**Figure 1:**
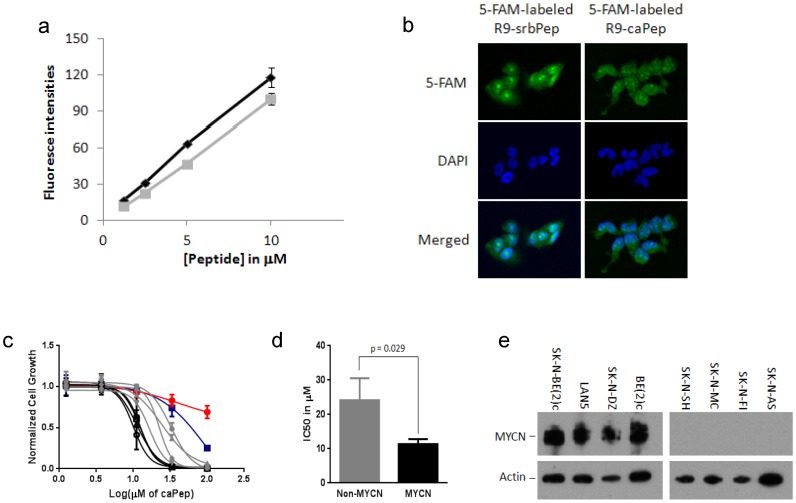
Permeability and selective cytotoxicity of R9-caPep in NB cells. a) SK-N-DZ NB cells were treated in triplicates by various concentrations of 5-FAM labeled R9-caPep (gray) or R9-srbPep (dark). After cells were treated by trypsin and washed, their fluorescence intensities were determined by flow cytometry. The median fluorescence intensities for triplicate cell populations under each treatment condition were averaged and graphed plus/minus standard deviations. b) Cells treated by 10 µM 5-FAM labeled R9-caPep or R9-srbPep were examined by confocal microscopy. The nuclear areas were indicated by DAPI staining. c) Four NB cell lines with *MYCN* amplification (in black), four NB cell lines without *MYCN* amplification (in grey), human PBMCs (red cycles), and human neural crest stem cell line 7SM0032 (blue squares) were cultured in the presence of various concentrations of the R9-caPep for 72 h. Cell growth was determined by a CellTiter-Glo luminescence assay (Promega). Cells cultured in the absence of the R9-caPep were used as control. Luminescent signals in triplicates normalized to the control for each cell line were averaged and graphed plus/minus standard deviations. Black filled squares: LAN5, black triangles: SK-N-DZ, black circles: SK-N-BE(2)c, black empty squares: BE(2)c, grey diamond: SK-N-AS, grey circle: SK-N-SH, grey triangle: SK-N-MC, and grey square: SK-N-FI. d) The IC_50_s of the peptide on cell lines with or without MYCN amplification, determined by non-linear fit of Prism 6 (GraphPad Software, La Jolla, CA), were averaged and graphed plus/minus standard deviations. e) Total cell lysates were extracted from the indicated cell lines. The expression of MYCN and actin in these cell lines were determined by western blot.

To determine the cytotoxic potential of blocking protein-protein interaction involving the L126-Y133 region of PCNA in cancer cells, we generated the R9-caPep by fusing the L126-Y133 sequence of PCNA to the C-terminus of a nine D-arginine sequence (R9) through a spacer of two cysteines. We also generated peptides R9-CC and R9-srbPep as controls (see Materials and Methods). To determine cell permeability of the peptides, we treated SK-N-DZ NB cells with various concentrations of 5-FAM labeled R9-caPep and R9-srbPep and measured their fluorescence intensity by flow cytometry. Quantification of the median fluorescence intensity of each cell population under various treatment conditions revealed that both peptides are cell permeable and are taken by cells with similar efficiencies in a dose dependent manner (Fig 1a). By fluorescence microscopy, we observed that R9-caPep and R9-srbPep localized to the cytosol, throughout the nucleoplasma, and likely in nucleoli (Fig 1b). They both formed small spots in the nucleoplasma with slightly different patterns presumably caused by their different affinities to nucleoproteins. R9-caPep inhibited the growth of a panel of NB cell lines with IC_50_ ranging from 10 to 32 µM (Fig 1c & d). In contrast, control peptides R9-srbPep and R9-CC did not significantly affect cell growth up to 50 µM (data not shown). In addition, R9-caPep was well tolerated by non-malignant cells including human peripheral blood mononuclear cells (PBMC) and human neural crest stem cells (Fig 1c) with IC_50_ of 98 µM and more than 100 µM on these cells respectively, indicating that the R9-caPep selectively inhibits the growth of NB cancer cells. Interestingly, NB cell lines with *MYCN* amplification were uniformly more sensitive to R9-caPep than NB cell lines without *MYCN* amplification (Fig 1c, d, & e). These observations demonstrate the potential utility of R9-caPep or R9-caPep derived agents for treating NB, especially the subset containing *MYCN* amplification associated with a particularly poor prognosis.

### The R9-caPep blocks PCNA interactions

**Figure 2:**
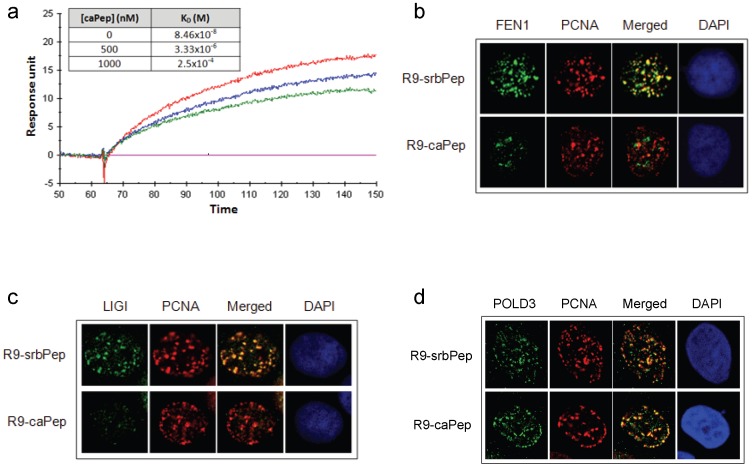
Inhibition of PCNA interactions by caPep and R9-caPep. a) The real-time SPR response curves were recorded for 1000 nM recombinant PCNA (rPCNA) flowing over the PIP-box sequence of FEN1 immobilized to the surface of a CM5 chip in the presence of 0 (red), 500 (blue), or 1000 (green) nM caPep. The dose-dependent binding of rPCNA to the immobilized FEN1 PIP-box sequence were also recorded under other rPCNA concentrations ranging between 250 and 1000 nM in the presence of 0, 500, or 1000 nM caPep (response curve not shown) and were used to calculate K_D_ of PCNA-FEN1 PIP-box interaction, as shown in the inserted table. SK-N-AS NB cells were treated with R9-caPep or R9-srbPep. Cells were fixed and immunostained with: b) mouse anti-FEN1 and goat anti-PCNA antibodies; c) mouse anti-LIGI and goat anti-PCNA antibodies; d) mouse anti-POLD3 and goat anti-PCNA antibodies. After DAPI counterstaining, nuclear co-localization of PCNA with FEN1, LIGI, or POLD3 was visualized by fluorescence confocal microscopy.

One essential function of PCNA is coordinating DNA replication by recruiting many interacting proteins, including FEN1, LIGI, and Pol ä, to replication foci. Binding of these proteins to PCNA not only brings them to the site of DNA replication, but also is often essential to their functional activity or processivity. To explore the mechanism by which R9-caPep kills cancer cells, we first examined whether the caPeptide (caPep) interferes with PCNA interaction *in vitro* by SPR (see Materials and Method for details). Given the fact that caPep is derived from the interdomain connecting loop of PCNA that usually contacts the PIP-box sequence in PCNA-binding proteins [11], [17], we tested whether caPep can block interaction between PCNA and the PIP-box sequence of FEN1. Shown in Fig 2a are the real-time response curves recorded for 1000 nM recombinant PCNA (rPCNA) flowing over the PIP-box sequence of FEN1 immobilized to the surface of a CM5 chip in the presence of 0, 500, or 1000 nM caPep. The presence of caPep significantly reduced rPCNA interaction with the immobilized FEN1 PIP-box sequence (Fig 2a). We also recorded binding curves under other rPCNA concentrations ranging between 250 and 1000 nM in the presence of 0, 500, or 1000 nM of caPep and observed that rPCNA binds to the immobilized FEN1 PIP-box sequence in a dose-dependent manner (data not shown). The dose-dependent PCNA binding to the immobilized FEN1 PIP-box sequence recorded under each caPep concentration was used for calculating dissociation constant (K_D_). As shown by the K_D_ in the inserted table in Fig 2a, caPep decrease the affinity of PCNA-FEN1 PIP-box interaction in a dose-dependent manner, indicating its antagonistic effect on PCNA interaction.

**Figure 3:**
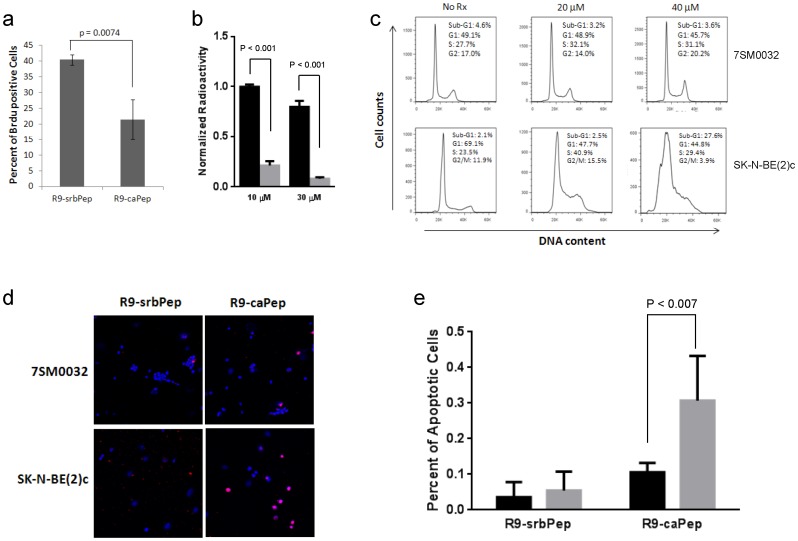
Inhibition of DNA replication and induction of S-phase arrest and apoptosis by R9-caPep. a) SK-N-BE(2)c cells were pulsed in 10 µM of BrdU for 30 min after being pre-treated with R9-caPep or R9-srbPep for 7.5 h. The relative abundances of BrdU-positive cells in triplicates were averaged and graphed plus/minus standard deviations. b) Nuclear extracts from SK-N-BE(2)c cells were incubated with the indicated concentrations of R9-caPep (grey bars) or R9-srbPep (black bars) for 20 min. SV40 T-antigen was then added to the nuclear extracts along with premixed reaction buffer containing ^32^P dCTP. A complete reaction mixture except for SV40 T-antigen was used as control for T-antigen-independent nucleotide incorporation. The polymerized radioactivity was measured by a scintillation counter. The T-antigen-dependent incorporation of ^32^P dCTP was calculated by subtracting T-antigen-independent radioactivity from the total radioactivity and was normalized to the T-antigen-dependent radioactivity in PBS-treated samples. Triplicates of normalized T-antigen-dependent radioactivity for each treatment condition were averaged and graphed plus/minus standard deviations. c) SK-N-BE(2)c and non-malignant 7SM0032 cells were fixed and stained with propidium iodide (PI) after being treated with the indicated concentrations of R9-caPeptide for 48 h. The cellular PI fluorescence intensity determined by flow cytometry was analyzed by the FlowJo to model various cell populations. d) Cells grown on chamber slides were treated by R9-caPep or R9-srbPep at 40 µM for 48 h. Cells were fixed and analyzed by a TUNEL assay. Cells were imaged by a confocal microscope. TMR-red is the fluorophore that was attached to the free DNA ends. DAPI (blue) indicates the location of nuclei. The pink dots derived from the merged TMR-red and DAPI staining indicate apoptosis. e) The abundance of apoptotic cells relative to the total number of cells in six randomly selected fields were averaged and graphed plus/minus standard deviations (right). The dark and gray bars represent results from 7SM0032 and SK-N-BE(2)c cells respectively.

We sought to determine whether R9-caPep treatment interferes with the interaction of PCNA with these proteins during DNA replication. Cells were synchronized by serum starvation followed by mimosine treatment (see Methods for details). We pre-determined that more than 60% of the cells were in the S-phase 6 h after mimosine was removed (data not shown). We treated synchronized cells with R9-caPep or R9-srbPep for 6 h as they were entering S-phase after being released from mimosine-induced growth arrest. Cell cycle analyses indicated that neither peptide significantly affected cell cycle progress within 6 h (data not shown) and a longer treatment increased the relative abundance of cells in S-phase (Fig. 3c). We determined the subcellular localization of PCNA, FEN1, LIGI, and POLD3 (the subunit of Pol ä that directly interacts with PCNA) by immunofluorescence microscopy. In cells treated with R9-srbPep, PCNA co-localized with FEN1 and LIGI at discrete foci (Fig 2b and c). The PCNA foci were also visible in cells treated with R9-caPep. However, LIGI co-localization to PCNA-positive foci was largely blocked by the R9-caPep (Fig 2c). In addition, the few remaining FEN1 foci in cells treated by the R9-caPep didn't appear to overlap the PCNA foci (Fig 2b). Since R9-caPep treatment didn't affect intracellular LIGI or FEN1 level in a western blot assay (data not shown), the lack of FEN1 or LIGI foci co-localized with PCNA indicated that R9-caPep interfered with the recruitment of FEN1 and LIGI to PCNA without dissociating PCNA from replication foci. Interestingly, R9-caPep did not seem to affect the recruitment of POLD3 to PCNA (Fig 2d), indicating a degree of selectivity in the R9-caPep effect on PNCA interactions.

### R9-caPep inhibits DNA replication and induces cancer cell cycle arrest and apoptosis

Because R9-caPep affects the PCNA interaction with FEN1 and LIGI, both of which are involved in the maturation of the lagging chain during DNA replication, we determined whether the peptide interferes with DNA replication in NB cells by determining the effect of R9-caPep on DNA synthesis using a BrdU incorporation assay. R9-caPep treatment induced a significant reduction in the percentage of BrdU-positive cells in comparison to the treatment by R9-srbPep (Fig 3a), indicating stalled DNA replication in cells treated with R9-caPep.

We further examined the effect of R9-caPep on SV40 T-antigen-dependent DNA replication *in vitro*. The SV40 viral system is a widely used model for studying eukaryotic DNA replication, partly because SV40 encodes only a single replication protein, the T antigen, and extensively utilizes cellular replication machinery [33]. As a result, the viral and eukaryotic DNA replications share remarkable resemblance. R9-caPep inhibited SV40 T-antigen-dependent DNA replication *in vitro* (Fig 3b), confirming the effect of R9-caPep on DNA replication observed in the BrdU incorporation assay. Consistent with these observations, R9-caPep treatment caused S-phase arrest in NB cells (Fig 3c). After 48 h of treatment by 40 µM of R9-caPep, NB cells start to die through apoptosis as indicated by the rise of a sub-G1 cell population (Fig 3c) and by increased TUNEL positivity (Fig 3d & e). In contrast, significantly less effect on cell cycle progression and cell survival were seen in non-malignant 7SM0032 cells under the same R9-caPep treatment (Fig 3c, d, & e). Collectively, these observations support the hypothesis that the R9-caPep exerts its effect on cancer cells at least partly by interfering with DNA replication through blocking PCNA interactions with its binding proteins.

### R9-caPep interferes with HR-mediated DSB repair

**Figure 4:**
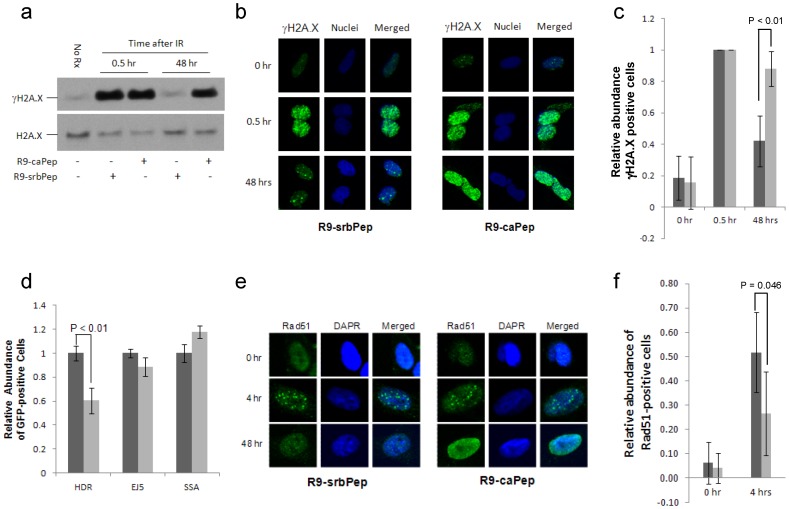
Inhibition of DSB repair by R9-caPep. Cells pretreated with 30 µM R9-caPep or R9-srbPep for 2 h were irradiated by a ã-irradiator (5 Gy). After irradiation, cells were cultured in the presence of R9-caPep or R9-srbPep for the indicated time. a) Intracellular ãH2A.X and total H2A.X levels were determined by western blot. b) The formation of intra-nuclear ãH2A.X foci was analyzed by immunofluorescence microscopy. c) Cells containing at least 5 ãH2A.X foci were counted as ãH2A.X positive cells. The relative abundance of ãH2A.X positive cells in five randomly selected fields were averaged and graphed plus/minus standard deviation. Dark bars represent results from cells treated with the scrambled R9-srbPep; Light bars represent results from cells treated with R9-caPep. d) The DR-GFP, EJ5-GFP, and SA-GFP cell lines were transiently transfected by the pCBASce plasmid that expresses the I-SceI meganuclease. The HR, EJ, and SSA-mediated DSB repair events, indicated by the restoration of a functional GFP gene in the respective cell lines, were quantified by measuring the relative abundance of GFP-positive cells by flow cytometry. The relative abundance of GFP-positive cells in R9-caPep treated samples (light bars) were normalized to those treated with R9-srbPep (dark bars). Results from triplets for each cell line and treatment condition were averaged and graphed plus/minus standard deviations. e) Cells pretreated with R9-caPep or R9-srbPep for 2 h were ã-irradiated (5 Gy). The formation of intra-nuclear Rad51 foci was analyzed at the indicated time after ã-irradiation by immunofluorescence microscopy. f) The relative abundance of Rad51-positive cells (containing at least 5 foci) as a percent of the total number of cells in five randomly selected fields were averaged and graphed plus/minus standard deviations. The dark and gray bars represent results from cells treated by R9-srbPep or R9-caPep respectively.

In addition to its role as the processivity clamp for the DNA replication machinery, PCNA also plays a broad role in repairing DNA damage including the lethal DSB [18]. To investigate whether R9-caPep treatment affects DNA DSB repair, we induced DSB by ã-irradiation of cells pre-treated with R9-caPep or R9-srbPep. Western blot analysis showed that the levels of ãH2A.X, a marker of double-stranded DNA damage, increased within 30 min following ã-irradiation in cells treated with either peptide (Fig 4a). In cells treated by the control R9-srbPep, the ãH2A.X level went down to a basal level by 48 h after ã-irradiation, suggesting the completion of DSB repair. In contrast, the ãH2A.X level remained elevated in cells treated by the R9-caPep, indicating the continued presence of unresolved DSB after 48 h. This result was further confirmed by immunofluorescence studies showing that R9-caPep treatment delayed the resolution of ãH2A.X foci induced by ã-irradiation (Fig 4b & c), indicating an impaired capacity for DSB repair in cells treated with R9-caPep.

Double-stranded DNA breaks, if not resolved in time, are lethal to cells. Cells deal with double-stranded DNA breaks through several DNA repair pathways, including HR, EJ, and SSA mediated DNA repair [34], [35]. Reporter cell lines have been established to monitor each of these DNA repair pathways [31]. These cells lines each contain a reporter with a GFP expression cassette disrupted by recognition site(s) for the endonuclease I-SceI. Introduction of exogenous I-SceI creates DSB(s) within the reporters. Each reporter is designed such that repair of the I-SceI-induced DSB(s) by a specific pathway can result in restoration of the GFP cassette: HR for DR-GFP, EJ for EJ5-GFP, and SSA for SA-GFP. The relative abundance of GFP-positive cells determined by flow cytometry, therefore, reflects the efficiency of the respective DSB repair pathway in these reporter cell lines. Using these characterized reporter cell lines, we observed that R9-caPep treatment significantly inhibited HR-mediated DNA repair, while causing only small effects on EJ or SSA (Fig 4d). The DSB repair event measured by DR-GFP (HR), but not EJ or SSA, is promoted by the recombinase Rad51 [30], which mediates strand exchange between sister chromatids. Accordingly, HR requires the recruitment of Rad51 to DNA damage, often measured as its accumulation into nuclear foci, which is dependent on BRCA1 and BRCA2 [36]. The formation of the Rad51-DNA recombination complex is known to be regulated by PCNA and its interacting proteins [37], [38]. We measured the effect of R9-caPep on the formation and/or resolution of Rad51 foci in response to ã-irradiation in SK-N-BE(2)c cells. Immunofluorescence microscopy indicated that R9-caPep treatment reduced the number of Rad51-positive cells at 4 h after ã-irradiation (Fig 4e and f). By 48 h after ã-irradiation, cells treated with the control R9-srbPep were able to almost completely resolve the Rad51 foci. In contrast, nearly all the cells treated with R9-caPep showed a strong and diffused background staining of Rad51, indicating an enhanced expression of Rad51. Over this strong background staining, Rad51 foci remain visible, suggesting that DNA repair following Rad51-DNA complex formation was blocked in R9-caPep treated cells.

### R9-caPep enhances the sensitivity of cancer cells to cisplatin

**Figure 5:**
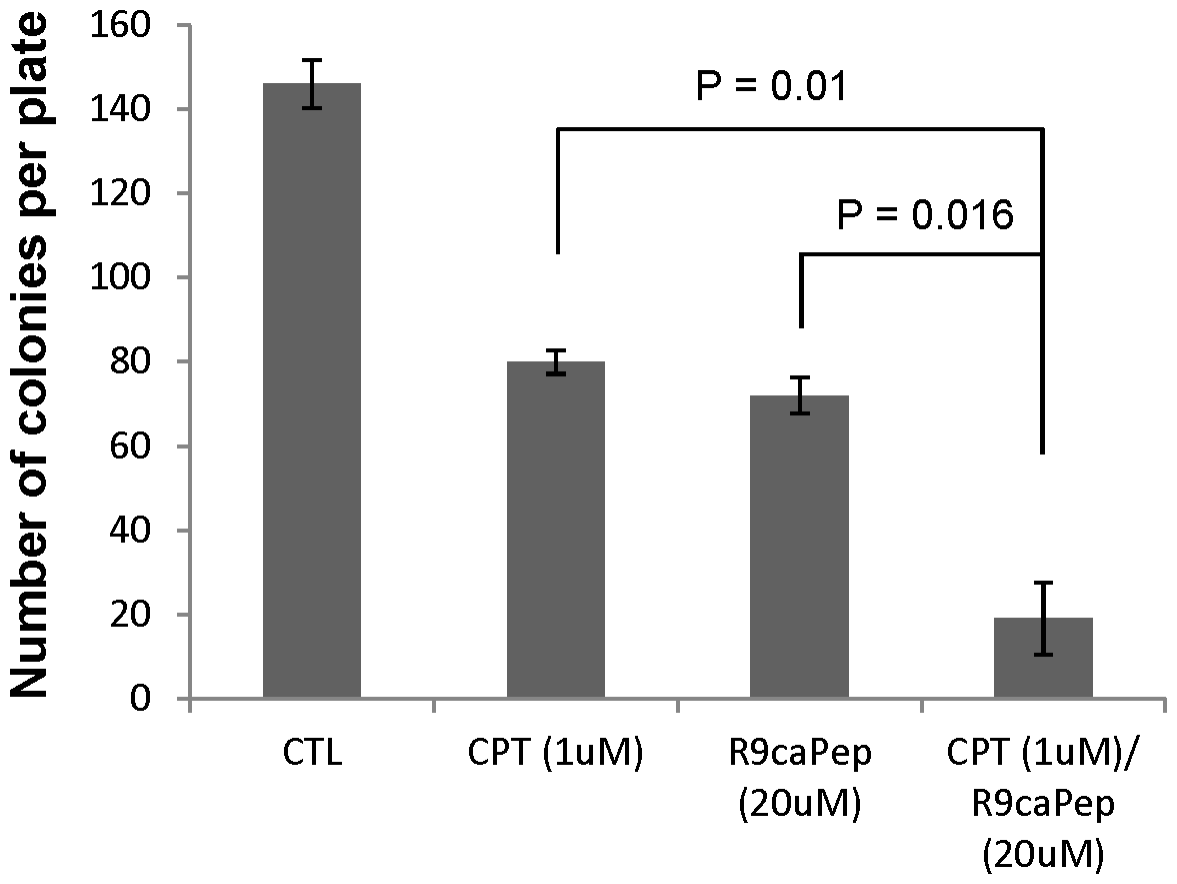
Enhanced sensitivity to cisplatin by R9-caPep. Human SK-N-BE2c NB cells were treated with or without 1 µM cisplatin for 2 h. Cells were washed twice with growth medium and were cultured in fresh medium with or without 20 µM R9-caPep for 3 weeks to allow colony formation. The colony counts in 3 dishes under each treatment condition were averaged and graphed plus/minus standard deviations.

HR-mediated DNA repair plays an important role in repairing cross-linked DNA [39], [40] caused by common chemotherapeutic drugs, such as cisplatin. We performed a clonogenic assay to investigate whether the peptide would increase NB cells' sensitivity to cisplatin. We treated SK-N-BE(2)c cells with or without 1 µM cisplatin for 2 h to induce DNA cross-linking. After being washed twice with growth medium, cells were cultured in fresh medium with or without 20 µM of R9-caPep in the absence of cisplatin for 3 weeks to allow colony formation. Whereas R9-caPep or cisplatin alone reduced the number of colonies formed by less than 30%, sequential treatment of cells with cisplatin and R9-caPep was able to reduce the number of colonies by about 80% (Fig 5), demonstrating the potential of combining R9-caPep-derived therapies with conventional chemotherapeutic drugs in treating NB patients.

### R9-caPep inhibits tumor growth in mice

**Figure 6:**
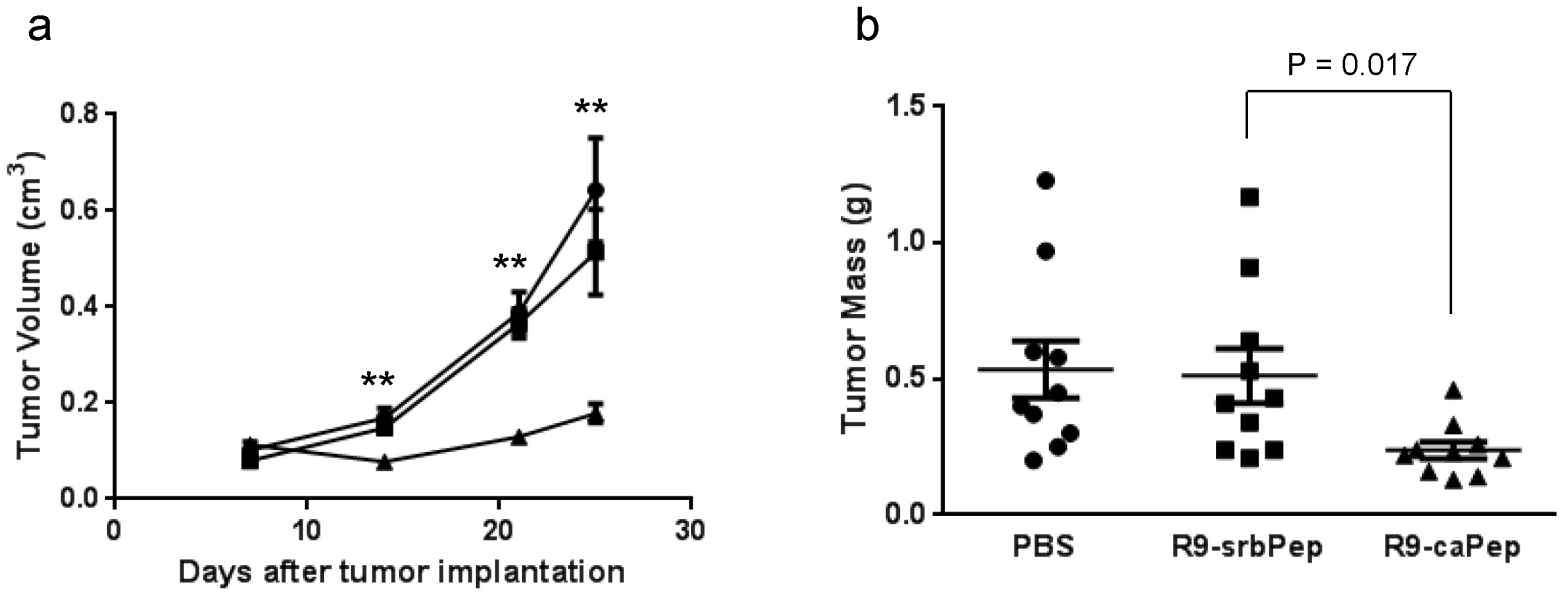
Inhibition of tumor growth by R9-caPep *in vivo*. a) Nude mice were randomly divided into 3 groups of 10 mice after each being injected with 5×106 SK-N-BE(2)c cells in Matrigel. Each group was treated with PBS (circle), R9-srbPep (square), or R9-caPep (triangle) by intratumoral injection. Tumor sizes were measured at the indicated time points and tumor volumes were estimated based on the length and width of the tumors (V = L×W2×0.5). The mean tumor volume for each treatment group was graphed plus/minus standard errors. ** indicates p<0.01. b) Tumor masses were measured at the end of the experiment and graphed in a scatter plot with mean plus/minus standard errors.

Given the favorable potential therapeutic properties of R9-caPep seen in cell-based assays, we asked whether we could recapitulate its anti-cancer activity *in vivo*. We tested R9-caPep in nude mice bearing xenograft tumors derived from the SK-N-BE2(c) cells and found that R9-caPep significantly and nearly completely inhibited tumor growth in terms of tumor volume and mass (Fig 6a & b) in comparison to the control groups that were treated with PBS or R9-srbPep. These *in vivo* results corroborate with our *in vitro* results and further suggest the potential of the PCNA L126-Y133 region in conceptualizing NB therapeutics.

## Discussion

Cancer cells depend on DNA replication as well as multiple DNA repair pathways to proliferate and survive. It is no coincidence that many chemotherapeutic agents act by damaging DNA or interfering with DNA replication or repair. These traditional chemotherapeutic drugs are widely used as first line therapies for treating a broad range of cancers, including NB. However, they are also toxic to normal cells, causing severe, debilitating side effects. In addition, there is a high risk of developing resistance to these drugs through mutations, resulting from the genetic instability characteristic of many cancers and redundancy in DNA synthesis and repair pathways. There is currently a considerable interest in the development of PCNA inhibitors as broad spectrum anti-cancer agents, because of the indispensible role of PCNA in regulating DNA replication and most DNA repair pathways. Proteins and peptides in general exhibit greater specificities to their targets than small molecules and are increasingly recognized as structural leads for the development of novel therapeutic small molecules. With the recent advancement in protein and peptide delivery technology, a number of protein and peptide based drugs have successfully reached markets or are currently working their way through different stages of clinical trials [41]. To target cancer cells selectively, we developed the therapeutic peptide, R9-caPep, which contains the L126-Y133 octapeptide region of PCNA and demonstrated that this peptide selectively kills NB cells and is especially toxic to *MYCN*-amplified NB cell lines, demonstrating its potential utility in treating patients with high-risk *MYCN*-amplified tumors.

Our work demonstrates that R9-caPep interfered with DNA replication and HR-mediated DNA repair presumably by binding to proteins that interact with PCNA through the L126-Y133 region, thereby preventing them from functionally being recruited to PCNA. Indeed, the caPep interfered with PCNA interaction with the PIP-box sequence of FEN1 *in vitro* and the R9-caPep blocked the co-localization of fen-1 and LIGI to the PCNA foci in cells undergoing DNA replication. Given the essential role these two proteins play in the processing of Okazaki fragments, the R9-caPep may stall replication of lagging strands by blocking Okazaki fragment maturation. The R9-caPep was also observed to block the repair of ã-irradiation-induced DSB. The effect of the R9-caPep on DSB repair appears to be specific to the HR-mediated pathway, since the peptide has little effect on the EJ pathway and slightly enhances DNA repair through SSA. The HR-mediated DNA repair is a major DSB repair mechanism in S and G2 phases and plays a vital role in resolving stalled replication forks [42]. When faced with DNA replication stress, cells attempt to overcome the stalled replication forks by transiently introducing DSB, which, if not resolved, is lethal to cells. By causing DNA replication stress and inhibiting HR-mediated DNA repair, the R9-caPep delivers a lethal one-two punch to cancer cells.

The most intriguing finding of this study is that NB cells with *MYCN* amplification are more sensitive to R9-caPep treatment than NB cells without *MYCN* amplification, as *MYCN*-amplified NB cancers are characteristically aggressive and resistant to therapy. We speculate that this phenomenon might be related to the dysregulated cell cycle control and DNA damage response in *MYCN*-amplified cells. *MYCN* is a member of the MYC proto-oncogene family that also comprises *MYC* and *MYCL*. It has been shown that MYC proteins promote the entry of S phase [43], [44] and inhibit G1 arrest after DNA damage [44]–[46]. Consequently, cells overexpressing MYC proteins are more likely to enter S-phase with unrepaired DNA damage. In the *MYCN*-amplified NB cell line, SK-N-BE(2)c, knockdown of MYCN expression by siRNA can restore DNA damage induced G1 arrest [44], indicating a causal relationship between MYCN overexpression and dysregulation of the G1 check point. In addition, both MYCN [47] and MYC [48] have been shown to directly induce DNA replication stress. Taken together, overexpression of MYC proteins likely makes cancer cells more dependent on HR-mediated DNA repair to resolve stalled DNA replication and, consequently, more sensitive to the blockade of DNA repair by R9-caPep. Consistent with this hypothesis, cancer cells overexpressing MYC proteins are addicted to DNA helicase, WRN, which plays an important role in resolving replication stress [43], [49]. The expression of a number of genes involved in the HR-mediated DSB repair pathway is also enhanced in NB tumors and cell lines containing *MYCN* amplification ([50] and data not shown). Given the structural similarity [51] and functional redundancy [52], [53] between MYCN and MYC, R9-caPep might be effective in treating cancers that overexpress MYC as well.

In addition to NB cells, the peptide is selectively toxic to breast, lung, and pancreatic cancer cell lines in comparison with non-malignant cell lines of their respective tissue origins (data not shown). The exact mechanism for such a specificity against a broad spectrum of malignancy remains to be elucidated. The interconnector domain of PCNA that contains the L126-Y133 sequence is a major binding site for many PCNA interacting proteins. Interestingly, whereas the R9-caPep blocked the co-localization of fen-1 and LIGI to the PCNA, it did not block PCNA and p21 interaction *in vitro* (data not shown) or the recruitment of POLD3 to PCNA foci in cells (Fig 2c). The differences in the ability of R9-caPep to block the recruitment of different PCNA interacting proteins may reflect the different affinities of these proteins to PCNA. It might also result from different binding affinities between the R9-caPep and these PCNA interacting proteins. We believe that the anti-cancer specificity of the R9-caPep is likely related to the unique profile of R9-caPep affinities towards various potential targets and their structural responses to R9-caPep binding. Identification of R9-caPep binding proteins by proteomic studies will be an important step toward understanding and further improving R9-caPep-type therapeutics. It has been shown that single amino acid substitutions in the L126-Y133 region can cause significant changes in the affinity profiles of PCNA for its interacting proteins in yeast [54]. Mutagenesis studies of the R9-caPep are ongoing to identify peptides with improved potency and therapeutic window. The structural and mechanistic insight gained from such studies will provide valuable information for the design of non-peptide mimetics of the R9-caPep. The fact that R9-caPep confers higher sensitivity to the DNA damaging agent cisplatin indicates its potential for combination therapy.
